# The Accessory Olfactory Bulb in *Arvicola scherman*: A Neuroanatomical Study in a Subterranean Mammal

**DOI:** 10.3390/ani14223285

**Published:** 2024-11-14

**Authors:** Sara Ruiz-Rubio, Irene Ortiz-Leal, Mateo V. Torres, Mostafa G. A. Elsayed, Aitor Somoano, Pablo Sanchez-Quinteiro

**Affiliations:** 1 Department of Anatomy, Animal Production and Clinical Veterinary Sciences, Faculty of Veterinary, University of Santiago de Compostela, Av. Carballo Calero s/n, 27002 Lugo, Spain; sara.ruiz.rubio@rai.usc.es (S.R.-R.); irene.ortiz.leal@usc.es (I.O.-L.); mateovazquez.torres@usc.es (M.V.T.); 2 Research Center for Molecular Medicine and Chronic Diseases (CIMUS), Instituto de Investigación Sanitaria (IDIS), University of Santiago de Compostela, 15705 Santiago de Compostela, Spain; 3 Department of Anatomy and Embryology, Faculty of Veterinary Medicine, Sohag University, Sohag 1646130, Egypt; mustafa.galal@vet.sohag.edu.eg; 4 Servicio Regional de Investigación y Desarrollo Agroalimentario (SERIDA), 33300 Villaviciosa, Spain; aitors@serida.org

**Keywords:** accessory olfactory bulb, vomeronasal system, fossorial water vole, immunohistochemistry, lectins, pheromones, kairomones

## Abstract

The accessory olfactory bulb (AOB) processes chemical signals crucial for species-specific socio-sexual behaviors. There is limited information about the AOB of wild rodents, and this study aims to characterize the neurochemical organization of the AOB in the fossorial water vole (*Arvicola scherman*), a subterranean Cricetidae rodent. We employed histological, immunohistochemical, and lectin-histochemical techniques. The AOB of these voles exhibits a distinct laminar organization, with prominent mitral cells and a dense population of periglomerular cells. Lectin histochemistry and G-protein immunohistochemistry confirmed the existence of an antero-posterior zonation. Immunohistochemical analysis demonstrated significant expression of PGP 9.5, suggesting its involvement in maintaining neuronal activity within the AOB. In contrast, the absence of SMI-32 labelling in the AOB, compared to its strong expression in the main olfactory bulb, highlights functional distinctions between these two olfactory subsystems. Calcium-binding proteins allowed the characterization of atypical sub-bulbar nuclei topographically related to the AOB. All these features suggest that the AOB of *Arvicola scherman* is adapted for enhanced processing of chemosensory signals, which may play a pivotal role in its subterranean lifestyle. Our results provide a foundation for future studies exploring the implications of these adaptations, including potential improvements in the management of these vole populations.

## Simple Summary

The accessory olfactory bulb (AOB) helps animals to process chemical signals, such as pheromones and kairomones, that are important for social and reproductive behaviors. This study focuses on the fossorial water vole, a rodent that lives mostly underground and relies on chemical communication. We examined the structure of the AOB in these voles using various staining techniques to visualize different cell types. The results showed that the fossorial water vole AOB has a well-organized structure, with specific cells in different layers. We also found that certain glycans and proteins are more active in the AOB compared to the main olfactory bulb, which suggests that these two areas of the brain have different roles in processing smells. This research helps us understand how the sense of smell of the fossorial water vole is specially adapted to its underground environment. The findings could be useful for further studies on how to manage these vole populations, as their behavior and chemical communication are essential for their survival and interactions in underground environments.

## 1. Introduction

In the past, olfaction was considered a singular system specializing in detecting volatile odor compounds [1]. Today, it is known that the olfactory system comprises several subsystems, each with distinct anatomical bases and functions [2,3]; there is also significant cooperation among these subsystems [4]. Among them, the vomeronasal system (VNS) stands out as responsible for chemical communication, namely the detection of a diverse range of chemical signals that mediate innate responses crucial for the coordination of species-specific social and reproductive behaviors [5,6]. Among these chemical signals, pheromones are prominent as molecules that trigger innate intraspecific behaviors, primarily sexual and reproductive behaviors. Kairomones also play a critical role, producing an interspecific aversive effect [7]. Additionally, the detection of major histocompatibility complex molecules by the VNS is crucial, as they guide individuals in mating decisions [8,9].

The VNS comprises a sensory receptor structure called the vomeronasal organ (VNO), which is internally lined by a neuroepithelium analogous to that of the olfactory system, yet distinct in its characteristics [10,11]. Vomeronasal receptors, initially identified as two major families, V1R and V2R [12,13], are located on the luminal surface of the epithelium, although other types of neuroreceptors, such as formyl peptide receptors, were subsequently characterized [14]. The neuroepithelium transmits information to the central nervous system via the vomeronasal nerves, specifically to a distinct area within the olfactory bulb, known as the accessory olfactory bulb (AOB) [15,16]. The presence of these neural projections is crucial for identifying the AOB, as this structure exhibits considerable morphological diversity among mammals [17]. Finally, the secondary projections of the VNS, predominantly to the vomeronasal amygdala, were demonstrated thanks to comprehensive tracer studies conducted in the 1970s [18,19]. The AOB is the main integrative neural center for the information detected by the VNO, as most of the sensory information collected by the VNO passes through the AOB [20]. However, it has been postulated that in rodents and lagomorphs, as well as in other groups of mammals, such as canids, there exists a projection of vomeronasal information to the transitional area between the main olfactory bulb (MOB) and the AOB, a region known as the olfactory limbus [21].

Additionally, the AOB in mammals presents a broad diversity, both from a macroscopic and microscopic anatomical standpoint [22,23]. This diversity requires, in certain mammalian groups, the use of histochemical and neurochemical techniques to precisely determine the presence of an AOB. This is the case with canids [16,24], mustelids [25], and even humans [26], for whom there are few available descriptions. In other species, however, the AOB occupies a very noticeable volume in relation to the main olfactory bulb. This is the case for rodents [27], marsupials [28], and lagomorphs [29], whose AOB is macroscopically evident. Ultimately, these differences imply that when characterizing the AOB of a species, extrapolations should be avoided, due to striking differences, even among closely related mammalian families, such as those in the order Chiroptera [30]. This fact becomes even more evident when considering the microscopic organization. Although, generally speaking, the AOB has a laminar structure analogous to that of the MOB, both the lamination and the pattern of glomerular organization, as well as the cellularity of each layer, vary significantly depending on the species [31,32]. Likewise, striking differences have been described in the organization of the projection cells, the mitral cells, which exhibit considerable diversity in their morphological and topographic patterns depending on the animal group.

Given the importance of chemical communication in rodents and the fact that rats and mice have been the most utilized and studied models in the characterization of the mammalian nervous system, there is extensive information regarding the AOB in laboratory rodents [33,34]. However, despite the large number of families included in the order of rodents, very few of them have been studied in terms of the microscopic and neurochemical organization of the AOB. To our knowledge, the AOB of wild rodents has only been studied in the capybara *Hydrochoerus hydrochaeris* [35,36], the degu *Octodon degus* [37,38], the ground squirrel *Spermophilus beecheyi* [38] and the beaver *Castor fiber* [39], species belonging respectively to the Caviidae, Octodontidae, Sciuridae, and Castoridae families, and their AOBs show notorious differences compared to those of laboratory rodents. In this study, we focus on the morphological and neurochemical characterization of the AOB of a rodent belonging to the Family Cricetidae, subfamily Arvicolinae, specifically the fossorial water vole *Arvicola scherman* (formerly *Arvicola terrestris* [40]), for which the information available on the olfactory subsystems is limited to the VNO [41]. As for the AOB of the Cricetids, to our knowledge, information only exists about the hamster *Mesocricetus auratus*, a domestic species of the subfamily Cricetinae [24,42,43,44].

Given that the fossorial water vole lives mainly underground in extensive burrow systems [45], it relies more on chemical senses than on physical senses such as hearing and vision [46]. Indeed, the comprehensive study of the species VNO, both morphologically and neurochemically [41], represents a first step towards confirming the importance of kairomone and pheromone detection. The study of the organization of the chemosensory systems of *Arvicola scherman* is of particular interest because it is a species that can spread and reach populations of up to 1000 voles/ha during population peaks in agroecosystems in the main mountainous areas of Europe, being considered a serious agricultural pest in much of its range [47]. This species is also considered a human health hazard, serving as a reservoir for zoonotic pathogens like *Leptospira* spp. [48] or *Borrelia burgdorferi* s.l. [49] as well as parasites such as *Toxoplasma gondii* [50] and *Echinococcus multilocularis* [51].

Kairomones are naturally released by predators and detected by their prey [52,53], causing an aversive reaction based on physiological changes such as an increase in blood cortisol levels, systemic stress, and notable behavioral alterations such as a change in habitat use [54,55,56]. This paves the way for the use of synthetic kairomone and pheromone analogs that could be employed in chemical-based strategies to improve integrated pest management in fossorial water voles [57,58]. An example of the feasibility of this approach is illustrated by the field study conducted by Poissenot et al. [59,60], based on the use of volatile organic compounds present in the urine and in the lateral scent gland (LSG) of conspecifics [61]. The success of such strategies underscores the necessity of an in-depth neuroanatomical and neurochemical understanding of the species, enabling more effective targeting and application of these compounds.

In this work, we address the neurochemical study of the AOB of the fossorial water vole using macroscopic and microscopic dissection techniques, conventional histological methods, primarily Nissl and hematoxylin staining, and immunohistochemical and lectin-histochemical techniques. This will provide the basis for further research to assess the physiological impact of kairomones on individual organisms, which could ultimately improve the integrated management of overabundant vole populations.

## 2. Materials and Methods

### 2.1. Specimen Collection

In total, 10 specimens of *Arvicola scherman*, with an equal representation of both sexes, were obtained from grasslands in the village of O Biduedo in the Municipality of Triacastela. The animals were caught using snap traps (Supercat^®^ Swissinno, St. Gallen, Switzerland) strategically positioned in burrow systems and left active for 24 h, with assistance from local farmers. These traps typically cause immediate death by head trauma. In instances where this outcome did not occur, cervical dislocation was applied swiftly, in line with the guidelines of Directive 2010/63/EU concerning the welfare of animals used for scientific purposes.

As the fossorial water vole is considered one of the most serious agricultural pests in grasslands and orchards in Spain [47], population control is mandated under Article 15 of Spanish Law 43/2002 on plant health (BOE 2008). The actions carried out in this study fall under the category of zootechnical purposes (Real Decreto 53/2013), thereby exempting the research from requiring ethical approval. Nevertheless, all procedures adhered to the principles outlined in European Directive 2010/63/EU for the protection of animals used in scientific work. Once captured, the specimens were transferred to the Anatomy Department at the Faculty of Veterinary Medicine for necropsy, with transportation times always kept under two hours.

### 2.2. Olfactory Bulb Extraction

To fully extract the brain, the mandible and eyes were first detached, followed by a longitudinal incision along the dorsal surface of the skull to enable the removal of the skin and underlying muscle layers. The cranial vault and nasal bones were carefully excised using gouge forceps, exposing the cerebral hemispheres and the olfactory bulbs. The next step involved delicately isolating the olfactory bulbs from their position within the ethmoidal fossa. These laminated structures are particularly fragile, and the olfactory and vomeronasal nerves, which traverse the cribriform plate of the ethmoid bone, anchor them tightly to the dura mater. The bony walls of the fossa were carefully removed, and the olfactory nerves were severed. Following this, the optic nerves, internal carotid arteries, pituitary stalk, and cranial nerves emerging from both sides of the brainstem were severed, freeing the brain. The entire brain was then immersed in freshly prepared Bouin’s fixative. This fixative is highly recommended for neuroanatomical studies due to its excellent tissue penetration and ability to preserve the integrity of soft, delicate structures, facilitating subsequent processing [62]. After 24 h of fixation, the specimens were transferred into 70% ethanol for storage.

### 2.3. Processing of Samples for Microscopic Study

#### 2.3.1. Paraffin Embedding

The process was carried out stepwise by submerging the samples sequentially in distilled water, followed by immersion in 70% ethanol, 90% ethanol, 96% ethanol, and finally pure ethanol (three washes). The tissues were subsequently clarified using a 1:1 ethanol–xylene solution, followed by two rinses in pure xylene. After this treatment, the tissue became permeable to paraffin infiltration, which was done at 60 °C for at least three hours. The sample was then encased in paraffin using a dispenser to create a solid block.

#### 2.3.2. Sectioning

Tissue sections were prepared using a rotary microtome set to a thickness of 8 µm. For each animal, the right olfactory bulb was entirely sectioned in a transverse plane, while the left one was sliced in a sagittal plane, with the latter providing a clearer and more comprehensive view of the laminar structure of the AOB. We used two individuals, one of each sex, for the histological series stained with hematoxylin and Nissl. Of the remaining eight, four were allocated to lectin studies and four to immunohistochemical studies, with two individuals of each sex in both cases.

### 2.4. General Histological Staining

To observe the various tissue components, hematoxylin-eosin (HE) was utilized as a general stain, while the Nissl stain was applied to highlight the soma and initial segments of neuronal processes. In all the specimens, the findings were consistent in terms of structural integrity and immunohistochemical patterns.

### 2.5. Immunohistochemical Staining

The initial step involved inhibiting endogenous peroxidase activity to ensure that the staining was specific to the primary antibody binding site. This was accomplished by incubating the slide in a 3% H_2_O_2_ solution in distilled water, which depleted endogenous peroxidase and rendered it inactive. Following three rinses in 0.1 M phosphate buffer (PB) at pH 7.2, the next step was to prevent non-specific bindings. For this purpose, a blocking serum was applied from the same species as the one used to produce the secondary antibody (ImmPRESS™ Anti-Rabbit and Anti-Mouse Kit, Vector Labs, Burlington, VT, USA). The blocking process was carried out at room temperature for at least 20 min.

The immunohistochemical analysis included a specific set of antibodies that provided valuable insights into the morphofunctional characteristics of the VNS. The data for each antibody used are presented in Table 1. Antibodies against Gαi2 and Gαo were utilized to identify which pheromone receptor families—V1R [63] or V2R [64], respectively—were expressed in the VNS. Mitral cells, the primary neural components of the AOB, were marked using antibodies against microtubule-associated protein 2 (MAP-2) [65] and SMI-32, a marker of neurofilament proteins expressed in the mitral cells of the OB in certain therian mammals [66]. Neuronal growth was assessed using antibodies against growth-associated protein 43 (GAP-43) [67]. The maturity of the system was evaluated with an antibody for olfactory marker protein (OMP) [68]. Calcium-binding proteins calbindin (CB), calretinin (CR), secretagogin (SG), and PGP 9.5 were employed to distinguish neuronal subpopulations [69,70,71]. Anti-DCX antibody targets doublecortin, a marker of immature neurons and neurogenesis [72].

Following an overnight incubation at 4 °C, the samples were rinsed three times in phosphate buffer (PB). Depending on the blocking agent applied, they were then incubated for 30 min with either ImmPRESS VR Polymer HRP Anti-Rabbit IgG or Anti-Mouse IgG reagents. Before proceeding to the visualization step, all samples were washed for 10 min in 0.2 M Tris-HCl buffer (pH 7.61). For visualization, a chromogen solution of 3,3-diaminobenzidine (DAB) was used. The reaction was developed using a 0.003% H_2_O_2_ and 0.05% DAB solution in 0.2 M Tris-HCl buffer, producing a brown precipitate. Negative controls were prepared by omitting the primary antibodies.

### 2.6. Lectin Histochemical Labelling

Lectin labelling, while similar to immunohistochemical techniques, relies on specific proteins known as lectins. These proteins have domains that recognize and bind non-covalently to terminal sugars in tissues, forming glycoconjugates. Unlike antibodies, lectins do not have an immune origin [73]. Lectins have been extensively used in the study of the olfactory bulb [74]. In this study, we employed five lectins (Table 2).

*Ulex europaeus* (UEA)

UEA specifically binds to terminal L-fucose, present in glycoproteins and glycolipids. It was chosen for this study because of its role as a specific marker for the AOB in several species, including mice [75], foxes [76], and wolves [32].

*Lycopersicum esculentum* (LEA)

LEA exhibits high affinity for N-acetylglucosamine and has been widely used as a comprehensive marker for both the main and accessory olfactory bulb in various species. These include rodents such as rats [77], mice [78], capybaras [36], and hamsters [42].

*Vicia villosa* (VVA)

VVA, which is isolated from fodder vetch, binds specifically to N-acetylgalactosamine structures [79]. In rats, it selectively stains the accessory olfactory bulb [80,81], but this is not the case in mice [82].

*Solanum tuberosum* (STA)

STA, which is isolated from potatoes, binds oligomers of N-acetylglucosamine and some oligosaccharides containing N-acetylglucosamine and N-acetylmuramic acid [83]. This lectin has previously been used in the characterization of the VNS in horses, bears, and dama gazelles [79,84,85].

*Dolichos biflorus* (DBA)

DBA is isolated from horse gram and has a carbohydrate specificity toward N-acetylgalactosamine [86]. DBA is an excellent marker for AOB neurons in species such as goats and sheep [87,88].

The lectin histochemical labelling process began with slides that had been deparaffinized and rehydrated. To suppress endogenous peroxidase activity, they were treated with a 3% H_2_O_2_ solution. A 2% bovine serum albumin (BSA) solution was applied to block non-specific bindings. The lectins were incubated overnight at 4 °C. Following this, the slides were exposed to a 90 min incubation in an avidin-biotin complex (ABC) solution with peroxidase (Vectorlabs, Burlingame, CA, USA). This complex interacts with the lectin during the incubation, enhancing the subsequent peroxidase reaction. The reaction was visualized using a solution of 0.003% H_2_O_2_ and 0.05% 3,3-diaminobenzidine (DAB) in 0.2 M Tris-HCl buffer, which produced a brown precipitate. Controls included tests without the lectin, as well as with lectins pre-absorbed with an excess of their corresponding sugars.

### 2.7. Image Acquisition

Digital images were acquired using an Olympus SC180 camera attached to an Olympus BX50 microscope (Tokyo, Japan). To create composite images from multiple photographs, PTGuiPro software version 12.27 (Rotterdam, The Netherlands) was employed due to the large size of the studied structures. Adobe Photoshop CS4 (San Jose, CA, USA) was utilized to modify brightness, contrast, and white balance levels; however, no enhancements, additions, or alterations to the image characteristics were performed.

## 3. Results

### 3.1. Macroscopic Study

**Figure 1:**
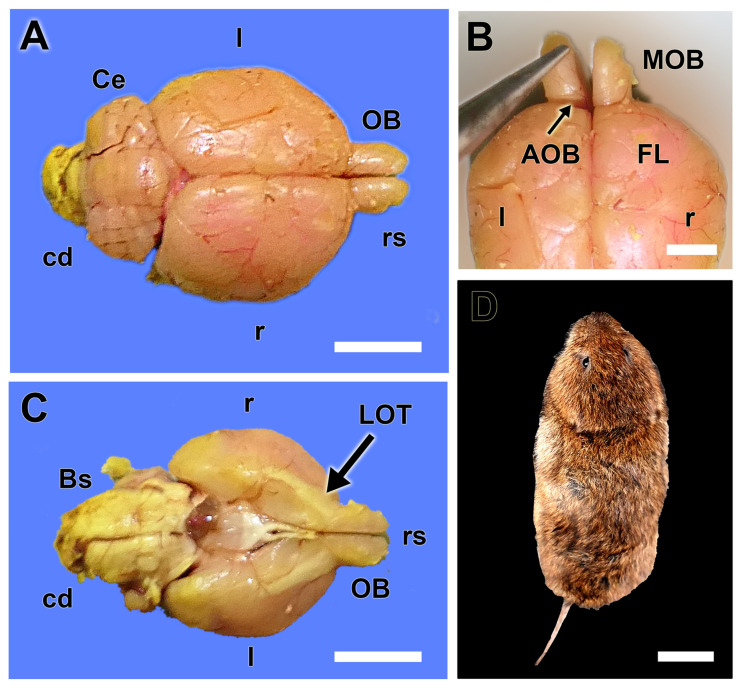
Macroscopic anatomy of the olfactory bulb of the fossorial water vole. (**A**) Dorsal view of the brain showing the relative size of the olfactory bulbs (OB). (**B**) Dorsal view of the rostral part of the telencephalon showing the location area of the AOB. (**C**) Ventral view of the brain showing the remarkable size of the lateral olfactory tract (LOT). (**D**) Dorsal view of one of the analyzed specimens of fossorial water vole. Bs: Brainstem; cd: Caudal; Ce: Cerebellum; FL: frontal lobe; l: left; MOB: main olfactory bulb; r: right; rs: Rostral. Scale bars: (**D**) = 2 cm, (**A**,**C**) = 1 cm, (**B**) = 0.5 cm.

The macroscopic study of the brain was conducted after its complete extraction (Figure 1), which was performed following the removal of the skin of the head and the dorsal opening of the cranial vault. The main olfactory bulbs exhibit a size and shape comparable to that of more extensively studied rodents, such as rats and mice. They are located at the rostral part of the brain, positioned anteriorly to the other structures, and are clearly visible as two projections extending from the telencephalon (Figure 1A–C).

The AOB is not easily recognizable macroscopically, as it does not form a prominent protrusion on the surface of the MOB and is also concealed by the frontal lobe of the telencephalon. Therefore, the best way to visualize it is by ventrally pulling the MOB with the aid of forceps. The ventral view of the brain allows for the observation of the remarkable relative size of the basal part of the rhinencephalon, which is associated with the olfactory subsystems (Figure 1C). There is a remarkable presence of a wide band of the lateral olfactory tract connecting the OB with the piriform lobe.

### 3.2. Histological Study

**Figure 2:**
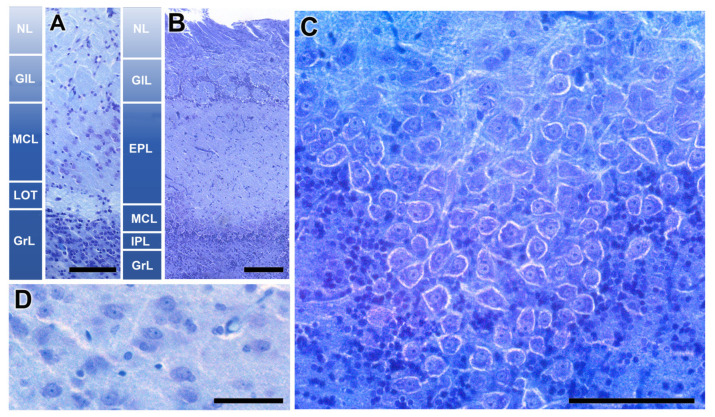
Histological structure of the olfactory bulb of the fossorial water vole. (**A**,**B**) Comparative study of the lamination of the accessory olfactory bulb and the main olfactory bulb, respectively. (**C**) Horizontal section of the mitral cell layer of the MOB. (**D**) Mitral cells of the AOB. Nissl Staining. EPL: Externa plexiform layer; GlL: Glomerular layer; GrL: Granular layer; IPL: Plexiform internal layer; LOT: Lateral olfactory tract; MCL: Mitral cell layer; NL: Nervous layer. Scale bars: (**A**–**C**) = 100 µm, (**D**) = 50 µm.

The histological study enabled the identification and exhaustive characterization of the accessory olfactory bulb of the fossorial water vole. It presents a well-defined laminar structure, similar to the main olfactory bulb, although there are remarkable differences between them, as shown in Figure 2A,B. The nervous layer of both structures receives vomeronasal and olfactory axons, respectively, for the AOB and MOB. The glomerular structures are clearly defined in both structures. They are the destination of the sensory axons and the synapsis point with dendrites of the subsequent neuron in the olfactory and vomeronasal pathway, the mitral cells. However, the MOB shows a much higher density of periglomerular cells.

The most remarkable difference is observed in the intermediate layers of the bulb, which contain the mitral cells. In the MOB, these are arranged along an axis forming an individualized lamina (MCL). In a horizontal section of this layer in the MOB, these cells exhibit a large size, with an atypical configuration not seen in other mammalian species (Figure 2C). Above this layer, there is a wide neuropil made up of the MC dendrites, known as the external plexiform layer (EPL). Its deeper counterpart is a thin layer, the internal plexiform layer (IPL), containing the beginnings of the axons of these mitral cells. However, the organization of the second-order neurons in the vomeronasal pathway is very different. The neuronal somas are not mitral or polygonal in shape, but oval (Figure 2D), and they are not arranged in a continuous lamina but are dispersed, equivalent to the EPL of the MOB. Therefore, we adopt the term mitral-plexiform layer (MPL) to design the resulting layer from the fusion of the two plexiform layers and the mitral layer. Deeper to the mitral plexiform layer is a specific layer of the AOB, the one that forms the lateral olfactory tract (LOT). This consists of a cord of white matter formed by the axons of mitral cells from both the MOB and the AOB. In the deepest plane, a broad layer of granular cells (GrL) is present in both systems.

**Figure 3:**
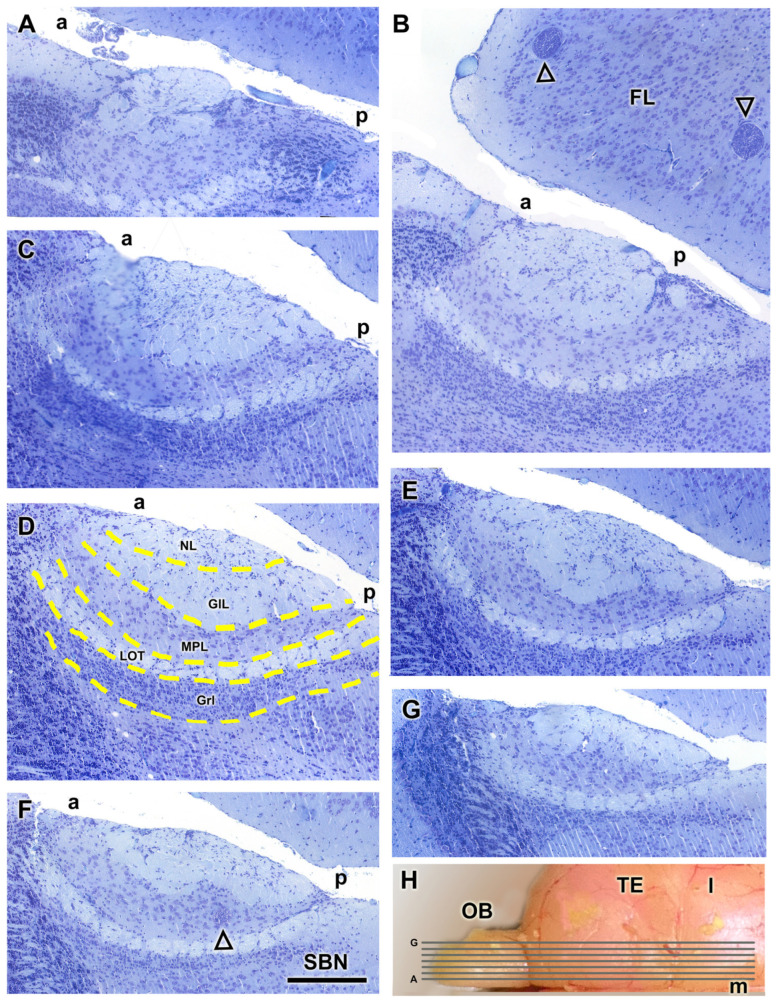
Nissl staining of sagittal serial sections of the accessory olfactory bulb of the fossorial water vole. (**A**–**G**) Correlative sections of the AOB arranged from medial to lateral. In (**D**), the layers of the AOB are outlined by a yellow dashed line. In (**B**), the open arrowhead represents parasitic cysts. (**E**,**F**) In the caudal sub-bulbar portion of the AOB, there is an accumulation of projection neurons, forming a structure we refer to as the sub-bulbar nucleus (SBN). (**H**) Dorsal view of the right hemisphere of the water vole showing the corresponding levels of sections (**A**–**G**). a, anterior; FL, frontal lobe; GlL; glomerular layer; GrL, granular layer; MPL, mitral plexiform layer; l, lateral; LOT, lateral olfactory tract; m, medial; NL, nerve layer; OB, olfactory bulb; p, posterior; TE, telencephalon. Scale bar = 250 µm.

The AOB was examined through Nissl-stained sagittal serial sections, to comprehensively characterize its laminar organization (Figure 3). The correlative sections illustrate from medial to lateral the structural organization of the AOB (Figure 3A–G). The glomerular, mitral-plexiform, and granular layers are clearly distinguishable, as outlined by a yellow dashed line in Figure 3D. Remarkably, the glomerular and mitral-plexiform layers exhibit a significant development. Incidentally, parasitic cysts, presumably caused by *Toxoplasma* spp., were identified in the frontal lobe (Figure 3B) and the MPL of the AOB (Figure 3E). The presence of such cysts is consistent with prior reports of parasitic infections in wild rodents. The lateral olfactory tract is visualized all through the AOB (Figure 3B–G).

### 3.3. Lectin Histochemical Study

**Figure 4:**
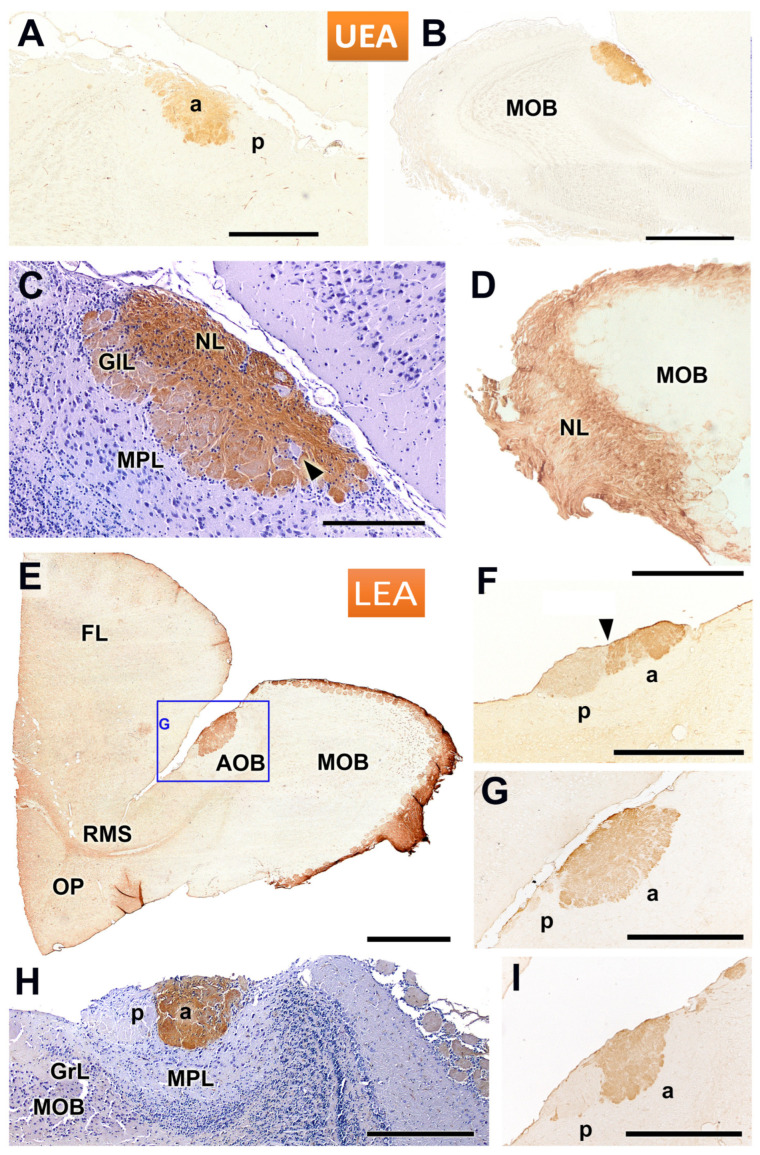
Lectin staining of the accessory and main olfactory bulbs in the fossorial water vole using UEA and LEA. (**A**–**D**) UEA staining allows for the identification of the nerve and glomerular layers of the accessory olfactory bulb (AOB). (**A**) Sagittal section shows the presence of a rostro-caudal zonation in the AOB, with a positive anterior portion and a negative posterior portion. This zonation does not extend to all sagittal planes of the AOB, as shown in images (**B**,**C**). A hematoxylin counterstained section (**C**) shows how some isolated AOB glomeruli remain unstained by the lectin (arrowhead). Depending on the specimen studied, there are significant differences in the staining pattern of the MOB. Some individuals are UEA-negative for the main olfactory bulb (MOB) (**A**,**B**), while others exhibit a positive pattern (**D**). (**E**–**I**) LEA staining consistently showed a positive pattern in the superficial layers of both the AOB and MOB. (**E**) Overview of the anterior telencephalon, showing strong LEA staining in the AOB (highlighted in (**G**)) and the MOB, along with the rostral migratory stream (RMS). (**F**,**G**,**I**) Sagittal sections of the same AOB at different levels reveal an anterior-posterior zonation (arrowhead in (**F**)). The intensity of the zonation depends on the level considered. (**H**) Counterstained image from a different individual confirming the zonation and showing how the staining encompasses the glomerular (GlL) and nerve (NL) layers of the anterior AOB. a, anterior; FL, frontal lobe; GrL, granular layer; MPL, mitral-plexiform layer; OP, olfactory peduncle; p, posterior. Scale bars: (**B**,**E**) = 1 mm; (**A**,**D**–**G**,**I**) = 500 µm; (**C**,**H**) = 250 µm.

Lectin staining of the AOB and MOB using UEA and LEA revealed distinct labelling patterns across different layers and regions. UEA staining enabled the identification of the superficial nerve and glomerular layers of the AOB (Figure 4A–D). A clear rostro-caudal zonation was observed in the AOB, with the anterior portion showing strong positive staining, while the posterior portion remained negative (Figure 4A). However, this zonation was not consistent across all sagittal planes of the AOB, as demonstrated in Figure 4B, where the anterior-posterior zonation is not visible. However, a hematoxylin counterstained section shows how some isolated AOB glomeruli remain unstained by the lectin (Figure 4C). In addition to variability in AOB staining, differences were observed in the staining pattern of the MOB depending on the specimen. This lectin histochemical staining was conducted on three individuals: in two of them, one male and one female, we observed staining only in the AOB across all sections studied (Figure 4A,B), while in a third male, positive staining was detected in both the MOB and the AOB (Figure 4D).

LEA staining consistently revealed a positive labelling pattern in the superficial layers of both the AOB and MOB in all cases examined. An overview of the anterior telencephalon showed robust LEA staining in the superficial layers of both the AOB and MOB, as well as in the rostral migratory stream (RMS) (Figure 4E). Sagittal sections of the AOB at different rostro-caudal levels revealed an anterior-posterior zonation in the LEA staining, although the degree of this zonation varied depending on the specific level observed (Figure 4F,G,I). A counterstained image from a different individual confirmed the presence of this zonation, with staining encompassing both the glomerular (GlL) and nerve (NL) layers in the anterior AOB (Figure 4H).

**Figure 5:**
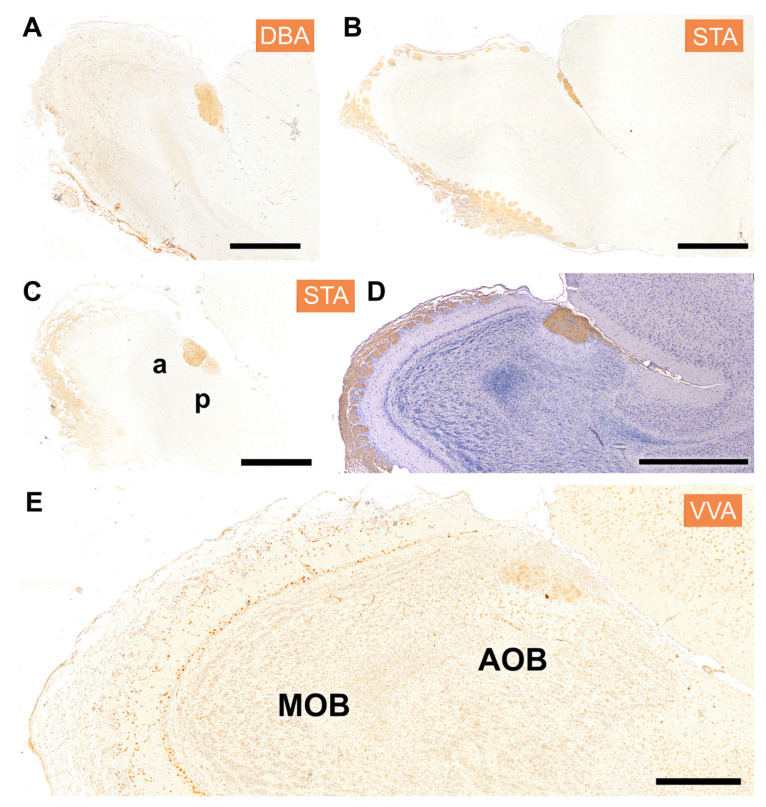
Histochemical staining of the fossorial water vole OB with the lectins DBA, STA, and VVA. (**A**) DBA specifically stains the whole AOB; it does not stain the MOB. (**B**–**D**) STA shows a similar staining pattern to that observed with the lectin LEA, with strong staining in the superficial layers of both the main and accessory OB. (**B**,**D**) correspond to two sagittal levels of the same individual, and (**C**) to a second specimen. Both individuals show anteroposterior zonation (**C**,**D**). (**E**) The lectin VVA shows generalized nuclear staining, mostly concentrated in the mitral plexiform and granular layer but involving a similar pattern to the MOB. a, anterior; p, posterior. Scale bar: (**A**–**D**) = 1 mm; (**E**) = 500 µm.

The distribution of lectin binding in the olfactory bulb (OB) with lectins DBA, STA, and VVA is shown in Figure 5. DBA staining was localized exclusively to the AOB, demonstrating a clear delineation between the AOB and the MOB, which remained unstained (Figure 5A). STA lectin resulted in a staining pattern that resembled that observed with LEA, prominently staining the superficial layers of both olfactory bulbs (Figure 5B–D). The figures shown correspond to two individuals, one of them counterstained with hematoxylin (Figure 5C). Both specimens confirmed the presence of anteroposterior zonation within the AOB (Figure 5C,D). Finally, VVA lectin exhibited generalized nuclear staining within the OB; in the AOB, this was predominantly concentrated in its mitral plexiform and granular layers (Figure 5E).

### 3.4. Immunohistochemical Study

**Figure 6:**
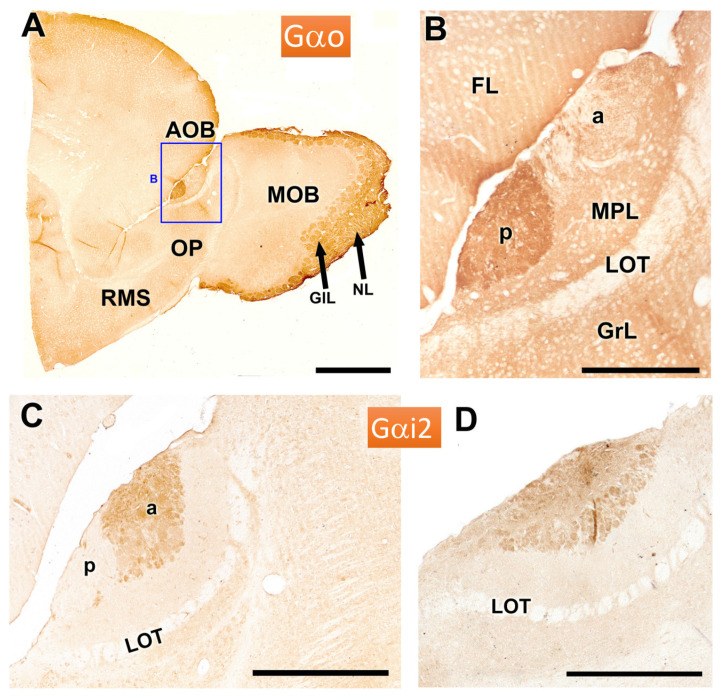
Immunohistochemical labelling of the AOB of the fossorial water vole with antibodies against the G protein subunits αo and αi2. (**A**,**B**) Immunolabelling with anti-Gαo. The anterior part of the telencephalon shows intense immunopositivity in the posterior zone of the AOB (enlarged in (**B**)) and in the superficial layers of the MOB. (**C**,**D**) Immunolabelling with anti-Gαi2 in two sagittal sections of the AOB shows anteroposterior zonation only in one of them (**C**), with the immunolabelling concentrated in the anterior part of the AOB. The more lateral section (**D**), however, does not show such zonation. a: anterior; FL, frontal lobe; GlL: Glomerular layer; GrL: Granular layer; LOT: lateral olfactory tract; MPL: Mitral plexiform layer; NL: Nervous layer; p: posterior; OP: Olfactory peduncle; RMS: rostral migratory stream. Scale bars: (**A**) = 1 mm, (**C**) = 500 µm, (**B,D**) = 250 µm.

The immunohistochemical study allowed for a deeper understanding of the morphofunctional characterization of the VNO. The study of the protein subunits of G protein, Gα0 and Gαi2, is crucial due to their direct relationship with the expression of the vomeronasal receptors of the V1R and V2R families. Gαo proteins are primarily associated with the V2R family, while Gαi2 proteins are related to the V1R family. In the AOB of the fossorial water vole, immunolabelling with anti-Gαo antibody shows a strong immunostaining in the posterior zone of the AOB, as well as in the superficial layers of the MOB (Figure 6A). The enlarged view of the AOB shows a clear demarcation from anterior and posterior regions of the AOB at the level of the superficial layers, nervous and glomerular (Figure 6B).

Immunolabelling with anti-Gαi2 reveals a complementary anteroposterior zonation to that observed with anti-Gαo, with the immunolabelling in this case concentrated in the anterior part of the AOB (Figure 6C). In contrast, a more lateral section does not exhibit such zonation, highlighting variability in the expression across the sagittal plane of the AOB (Figure 6D).

**Figure 7:**
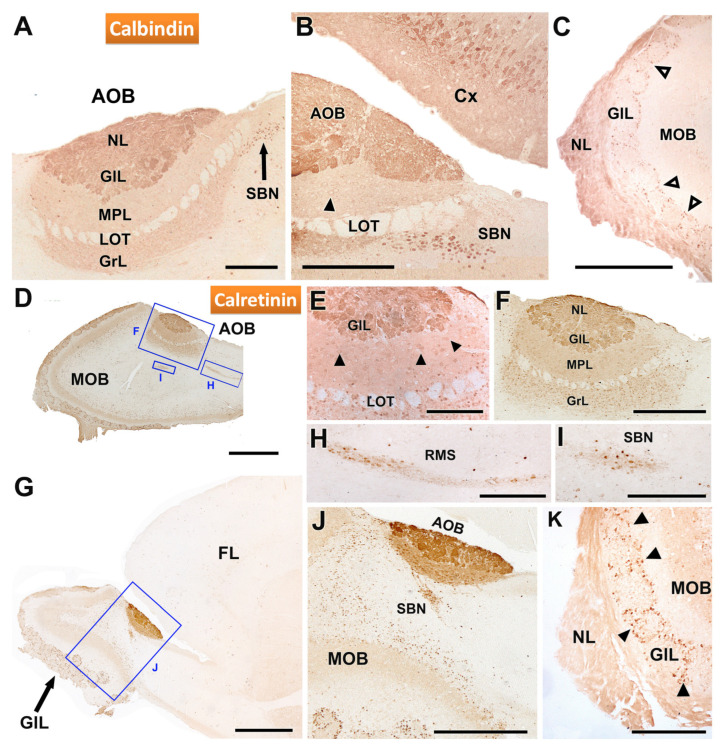
Immunohistochemical labelling of the AOB of the fossorial water vole with antibodies against the calcium binding protein calbindin (CB) and calretinin (CR). (**A**–**C**) Immunostaining with anti-CB. (**A**) Immunostaining in the AOB is concentrated in the nerve and glomerular layers, although the mitral plexiform and granular layers are also positive but with a weaker signal. Additionally, a nucleus of immunopositive neurons is identified in a sub-bulbar position (SBN). (**B**) Enlargement of the caudal area of the AOB shown in (**A**). The neuronal somas of the SBN and the pyramidal neurons of the cerebral cortex (Cx) show strong immunopositivity. The mitral cells of the AOB (arrowheads) are weakly stained. (**C**) The MOB shows intense staining in the nerve layer, with no staining of the glomeruli but marking of the periglomerular cells (open arrowheads). (**D**–**K**) Anti-CR immunostaining. (**D**) Sagittal section of the OB showing immunopositivity in the AOB and MOB. (**E**) Anti-CR produces intense staining in the nerve and glomerular layers as well as in the mitral cells of the AOB (arrowhead). (**F**) Enlargement of the inset in C showing immunostaining comprising all layers of the AOB, being especially intense in the two superficial layers, nerve (NL) and glomerular (GL). (**G**) Low-magnification sagittal view of the immunostaining in the anterior part of the telencephalon. (**H**,**I**) Enlargements of the boxes in G showing, respectively, the intense immunopositivity in the RMS and the SBN. (**J**) Enlargement of the box in G, showing the direct connection between the AOB and the RMS. (**K**) Immunostaining in the MOB extends to the nerve layer and the periglomerular cells (arrowhead). FL, frontal lobe; GlL, glomerular layer; GrL: granular layer; LOT, lateral olfactory tract; MPL, mitral-plexiform layer; NL, nerve layer; RMS: Rostral migratory stream. Scale bars: 1 mm = (**D**,**G**), 500 µm = (**J**), 250 µm = (**A**–**C**,**F**,**H**,**I**), 50 µm = (**E**).

Immunohistochemical analysis using antibodies against calbindin (CB) and calretinin (CR) revealed distinct patterns of labelling in the AOB and MOB (Figure 7). Anti-CB staining produced a strong labelling in the nerve (NL) and glomerular (GL) layers of the AOB. However, the mitral plexiform (MPL) and granular (GrL) layers also displayed positivity, albeit with a weaker intensity (Figure 7A). Additionally, a nucleus of strong immunopositive neurons was detected in the sub-bulbar region (SBN) (Figure 7B). In the MOB, CB immunostaining was particularly intense in the nerve layer, but the glomerular neuropil remained unstained. Nevertheless, periglomerular cells showed strong immunopositivity (Figure 7C).

Immunohistochemistry with anti-calretinin further clarified the labelling patterns in both the AOB and MOB (Figure 7D–K). Both structures showed strong immunopositivity, with strong labelling in the nerve, glomerular, and granular layers of the AOB (Figure 7D–G). Mitral cells of the AOB were also immunolabelled with anti-CR (Figure 7E). Examination of the AOB at higher magnification revealed that immunostaining extended to all layers, though it was particularly intense in the superficial layers (NL and GL) (Figure 7E,F). A low-magnification sagittal view of the anterior telencephalon (Figure 7G) revealed calretinin immunoreactivity in the rostral migratory stream (Figure 7H) and in the SBN (Figure 7I). Remarkably, a direct topographical relationship between the AOB and the RMS was evident (Figure 7J). In the MOB, calretinin staining was prominent in the nerve layer and in periglomerular cells (Figure 7K).

**Figure 8:**
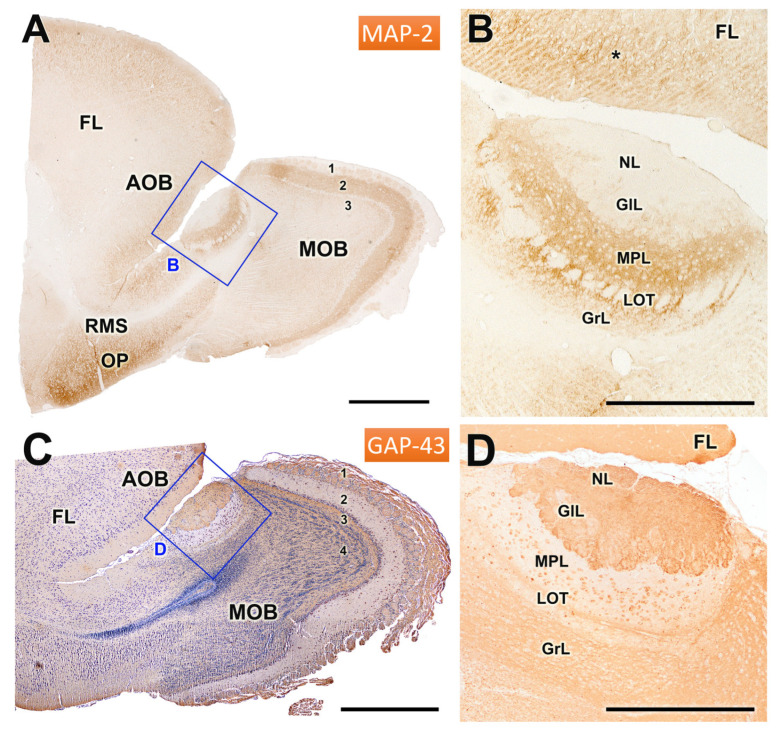
Immunohistochemical labelling of the olfactory bulb of the mole rat with anti-MAP2 and anti-GAP43. (**A**,**B**) Immunostaining of the olfactory bulb with anti-MAP2. (**A**) Sagittal section of the anterior part of the telencephalon, showing strong immunostaining in the external plexiform layer of the MOB (2), the mitral-plexiform layer (MPL) of the AOB, and the olfactory peduncle (OP). The rostral migratory stream (RMS) is immunonegative. (**B**) Enlargement of the inset in (**A**), showing immunostaining in both the AOB and the frontal lobe (*). The dendrites of the mitral-plexiform (MPL) and granular layer (GrL) are strongly stained; however, the cellular somas are immunonegative. (**C**,**D**) Immunostaining of the OB with anti-GAP43. (**C**) H-E counterstained sagittal section of the rostral telencephalon. The pattern of anti-GAP43 immunostaining is complementary to that observed with anti-MAP2. Immunopositivity is thus observed in the superficial layers of the AOB and MOB (NL and GlL) and in the GrL of AOB and MOB. (**D**) Higher magnifications of the inset in (**C**) show strong immunopositivity in the superficial layers; additionally, the mitral cells of the MPL layer and the lateral olfactory tract (LOT) traversing it are also immunopositive. FL: Frontal lobe; 1: Nerve + glomerular layer; 2: External plexiform layer; 3: Internal plexiform layer; 4: Granular layer. Scale bar: (**A**,**C**) = 1 mm; (**B**,**D**) = 500 µm.

Immunohistochemical analyses using antibodies against MAP2 and GAP43 are depicted in Figure 8A,B. Anti-MAP2 produced strong immunoreactivity in the external plexiform layer of the MOB and in the mitral-plexiform layer of the AOB, as well as in the olfactory peduncle (Figure 8A) and the frontal lobe (Figure 8B). Remarkably, the rostral migratory stream (Figure 8A) and the lateral olfactory tract (Figure 8B) exhibited no staining, indicating a lack of MAP2 expression in this area (Figure 8A). Within the AOB, the dendrites of MPL and GrL displayed strong immunoreactivity, whereas the cellular somas remained unstained, suggesting differential localization of MAP2 within neuronal components.

The staining pattern with anti-GAP43 (Figure 8C,D) showed a complementary pattern to anti-MAP2 immunostaining. Thus, the immunopositivity was primarily concentrated in the nerve, glomerular, and granular layers of both the AOB and MOB (Figure 8C). Additionally, in the AOB, the mitral cells within the MPL layer and the lateral olfactory tract traversing them also displayed strong immunopositivity for GAP43.

**Figure 9:**
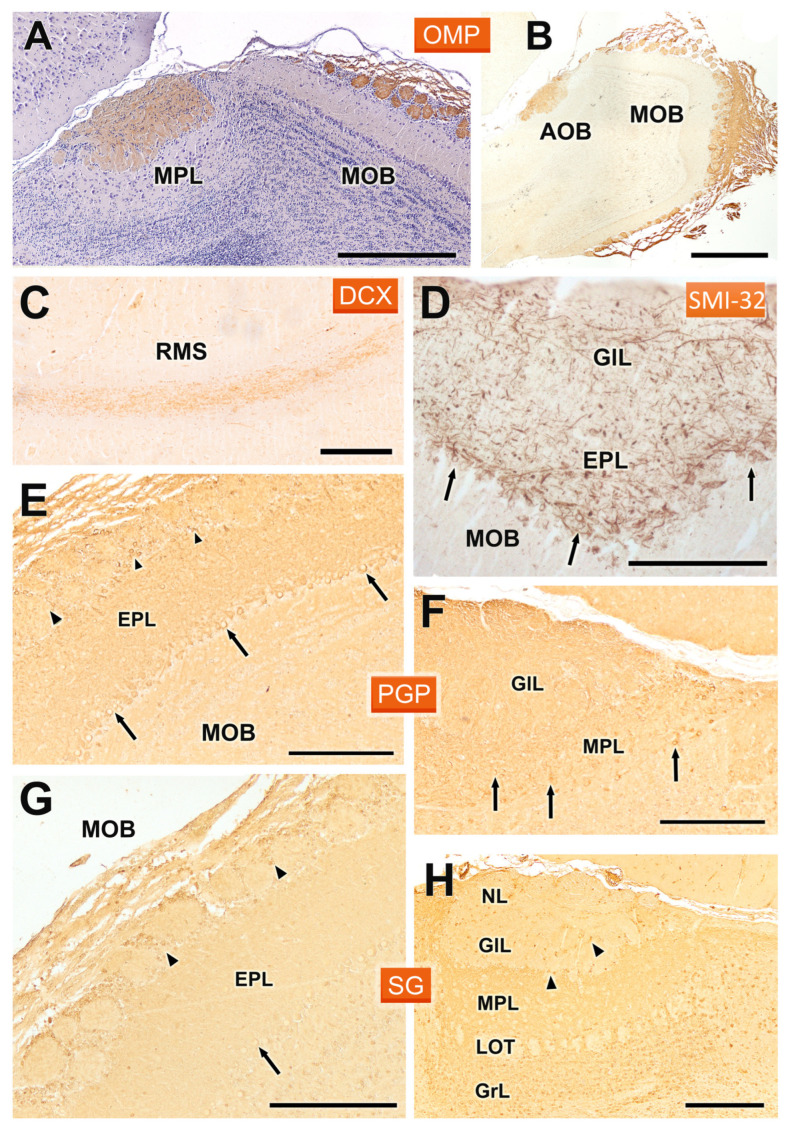
Immunohistochemical labelling of the fossorial water vole OB with antibodies against OMP, DCX, SMI32, PGP, and SG. (**A**,**B**) Anti-OMP produces intense labelling in the superficial layers of the AOB and MOB. The hematoxylin counterstained image (**A**) shows the absence of anterior-posterior zonation. (**C**) Anti-DCX labels the cells of the RMS. (**D**) Anti-SMI32 only stains the MOB, where it intensely labels the somas (arrows) and dendrites in the mitral and external plexiform layer (EPL). (**E**,**F**) Anti-PGP produces generalized labelling of the neuropil in both the MOB and AOB. At the cellular level, in the MOB (**E**) it labels the mitral (arrows) and periglomerular cells (arrowheads), whereas in the AOB (**F**) it produces a similar pattern to that observed in the MOB but without labelling the periglomerular cells (arrows, mitral cells). (**G**,**H**) Anti-SG produces a similar pattern in both structures, the MOB (**G**) and AOB (**H**), although it does not stain mitral cells in the latter (arrowheads, periglomerular cells; arrows, mitral cells). GlL: Glomerular layer; GrL: Granular layer; LOT: Lateral olfactory tract; MPL: Mitral-plexiform layer; NL: Nerve layer. Scale bars: 1 mm = (**B**), 500 µm = (**A**), 250 µm = (**C**–**H**).

We completed the immunohistochemical analysis of the OB with antibodies against OMP, DCX, SMI32, PGP, and SG (Figure 9). Anti-olfactory marker protein (Figure 9A,B) produced a homogeneous labelling of the superficial layers of both the AOB and MOB. Thus, there was no evidence of anterior-posterior zonation. The intensity of the immunostaining was higher in the AOB than in the MOB. Anti-DCX demonstrated the presence of doublecortin in the cells belonging to the rostral migratory stream, which suggests active neurogenesis and a dynamic movement of new neurons from the subventricular zone towards the OB (Figure 9C). In contrast, anti-SMI32 immunostaining was localized specifically in the MOB, with intense immunoreactivity observed in the somas and dendrites of neurons within the mitral and the external plexiform layer (Figure 9D).

The staining with anti-PGP (Figure 9E,F) and anti-SG (Figure 9G,H) showed very similar patterns in both the MOB and AOB. In both cases, there was generalized labelling of the neuropil, reflecting widespread neuronal activity in these regions. In the MOB, anti-PGP strongly labelled mitral cells and periglomerular cells (Figure 9E), while in the AOB a similar pattern was observed, though the periglomerular cells were not stained (Figure 9F). Similarly, anti-SG also labelled the neuropil in both structures, although mitral cells in the AOB did not show immunoreactivity, which may suggest differences in synaptic organization and function between the MOB and AOB (Figure 9H).

## 4. Discussion

One of the major limitations in the study of chemical communication in mammals is the scarce information available about their chemosensory systems, particularly the vomeronasal systems. This challenge is further complicated by the high morphological variability of the vomeronasal system among different mammalian groups, which makes the extrapolation of findings difficult [89]. If the available data on the structure and, especially, the neurochemistry of the VNO are limited, the situation becomes even more challenging when addressing the AOB [90]. In rodents, for example, most studies are restricted to laboratory rodents, with only a limited number focusing on other rodent groups, in few instances addressing not only the classical morphological characteristics but also neurochemical expression as well as the role of glycoconjugates as identified through lectin binding.

For this reason, we chose to focus our attention on the AOB of the fossorial water vole, a subterranean rodent species belonging to the family Cricetidae, which probably relies on its chemical senses for survival [45]. This study aims to bridge the gaps in our understanding of the VNS in wild rodents by providing a comprehensive characterization of the AOB using both routine histological techniques, such as Nissl staining, and more specific tools like immunohistochemical markers and lectin-histochemical staining.

### 4.1. Histological Study

One of the key findings of this study is the well-organized laminar structure of the AOB, which resembles that of the MOB but displays distinctive features. The arrangement of mitral cells in the AOB is less regular than in the MOB, as seen in other rodent species, such as hamsters [43], capybaras [36] and octodons [37]. This irregular organization, combined with the fusion of the mitral and plexiform layers, likely reflects specific processing requirements for vomeronasal input, highlighting the functional specialization of the AOB in detecting chemosignals [23]. The AOB of the fossorial water vole rat is also remarkable for the thickness of all its constitutive layers: nervous, glomerular, mitral-plexiform, and granular, as well as the lateral olfactory tract that traverses it. The mitral-plexiform layer is distinguished by the conspicuous number of principal cells that span the layer, consistently observed across the histological series, from the medial to lateral sections. Equally remarkable is the arrangement of the mitral layer in the MOB, characterized by a high density of principal neurons with large somas. Previous research indicates that olfactory stimulation can enhance both the area and density of mitral cells [91]. Therefore, these morphological features are likely indicative of the heightened olfactory activity typical of this species.

An incidental finding in some of the studied individuals was the presence of parasitic cysts in the frontal lobe of the brain and, in some cases, even in the MPL layer of the AOB. This is significant not only because it highlights the role of the species as a vector for diseases, such as toxoplasmosis, but also because these cysts can cause behavioral changes in the individual, leading to increased vulnerability to predators that are part of the parasites’ trophic chain [92].

### 4.2. Lectin Histochemical Study

The study of the olfactory bulb (OB) with lectins is of particular interest, as these markers enable selective characterization of the olfactory pathways—either the vomeronasal or the main olfactory pathway separately or in conjunction—depending on the lectin used and the species involved [93]. Moreover, lectin histochemical labelling has facilitated the characterization of the anterior-posterior zonation of the AOB, which corresponds to the differential expression of G proteins ai2 and ao, which are respectively associated with the V1R and V2R vomeronasal chemoreceptor families [94]. This zonation has been well characterized in the laboratory mouse [82] and rat [95], and among wild rodents in the hamster [42], capybara [36], degu [35], and beaver [39]. In the case of the fossorial water vole, the validity of lectin labelling in revealing a clear antero-posterior zonation is confirmed. Out of the five lectins used, three of them, UEA, LEA, and STA, allowed its identification, whereas DBA and VVA, although they had different labelling patterns—DBA in superficial layers and VVA in the mitral-plexiform layer—in no case revealed the existence of zonation. These results coincide with the zonation observed with UEA in mice [75,96], rats [97], and wallabies [16], with LEA in mice [96] and STA in hamsters, although in the latter case it was much less noticeable [42].

Beyond zonation, each employed lectin presented unique patterns that were repeated in the AOB of all studied individuals. The only exception was UEA, a lectin specific to the L-fucose pathway, which, as characterized by Kondoh et al. [75] in mice, shows a very variable expression pattern among individuals, independent of age and sex. Thus, depending on the individual studied, UEA in some instances was specific to the AOB, while in others it stained both the accessory and main olfactory bulbs to varying degrees. We confirmed this observation in the study of the fossorial water vole, observing a similar variable pattern. Some authors suggest that these individual variations reflect rapid dynamic changes in the main and accessory olfactory bulbs, thereby causing alterations in marking patterns among individuals, regardless of sex or age [75,98]. Furthermore, this individual variation may explain the disparate results found with UEA lectin in other rodents such as the rat, where Barber [99] and Ichikawa [80] respectively considered UEA specific to the AOB or to the OB as a whole. A completely disparate case that exemplifies the great diversity in the expression of this lectin is the fact that UEA in rabbits does not recognize either the AOB or the MOB at all [29].

The study with LEA lectin exemplifies how special caution must be taken when assessing these individual differences. As a result of our histochemical staining along the entire series of the AOB in the sagittal plane, we found that the labelling pattern changes significantly. Depending on the area considered, areas are observed that are totally positive in the anterior–posterior axis. Others, mainly located in the central and lateral areas of the BOA, show clear zonation. This process is a reflection of the asymmetries found in the compartmentalization of the accessory olfactory bulb in other rodents, such as in the case of the degus [37]. Once again, this fact could explain the assumed absence of zonation with LEA lectin in the AOB of the rat [97] or rabbit [29] or the minimal differentiation observed by Taniguchi et al. in the hamster [42]. STA lectin has been less studied in the AOB, although the zonation observed by us in the water vole lends greater validity to the zonation detected by Ichikawa et al. in the rat [80] and Taniguchi et al. in the hamster [42]. DBA does not produce zonation but generates intense labelling in *Arvicola scherman*, which differentiates this species from the weak marking observed in mice [96], rats [97], and hamsters [42]. The peculiar labelling pattern produced by the VVA lectin in the fossorial water vole is striking, with a strong positivity focused in the mitral plexiform layer that contrasts with the weak labelling observed in the mouse by Salazar et al. [96] or in the rat by Takami et al. [81]. However, in this latter species, Salazar et al. [97] and Ichikawa et al. [80] found intense labelling in the AOB, as did Shapiro et al. in the opossum [100]. Finally, in the case of the hamster, Taniguchi et al. [42] found a broad labelling pattern. It is also remarkable that the strongest VVA lectin staining is observed at the intranuclear level rather than the cytoplasmic level. Although uncommon in lectins, nuclear staining by VVA and other lectins has been reported in the vomeronasal system of reptiles [101], indicating potential evolutionary parallels in nuclear O-GalNAc glycosylation across species. The study of O-GalNAc-type glycosylation within the cell nucleus is still limited, especially with respect to N-acetylgalactosamine [102]. However, it has been demonstrated that all elements required for initiating O-GalNAc glycan biosynthesis are indeed present in the nucleus [103]. Our findings in the AOB reveal that nuclear staining does not label all cells uniformly but instead marks subpopulations of mitral, granular, and periglomerular cells. This suggests a significant differential activation of the pathway responsible for nuclear glycosylation across different cellular compartments within the AOB and MOB, which may be associated with unique functional roles of these subpopulations in pheromonal olfactory processing.

Our results confirm the utility of lectin markers in revealing distinct and variable patterns within both the AOB and the MOB, suggesting that lectin binding patterns can serve as differential markers to decode the intricate structural and functional organization within these regions. The differences in lectin binding among species suggest that these markers can be useful tools for a better understanding of the olfactory mechanisms across different groups of mammals.

### 4.3. Immunohistochemical Study

The immunohistochemical study with antibodies against the αo and αi2 subunits of G proteins is of particular interest, as the presence of both subunits is directly linked to the effective expression of the two main families of vomeronasal receptors, V1R and V2R. Specifically, Gαi2 is present in the transduction pathway of V1R receptors, while Gαo is involved in that of V2R. The immunohistochemical study in the AOB confirmed the segregated projection of both vomeronasal receptor families, with V1R receptors concentrated in the anterior zone of the AOB and V2R in the posterior zone. This pattern has also been observed in laboratory rodents such as rats [63] and mice [94], as well as in wild rodent species such as the beaver [39] and capybara [36]. However, in the case of the squirrel, no similar zoning has been identified, as the only study conducted found an AOB that exclusively expresses Gαi2 [38].

The anteroposterior zonation of the AOB has been correlated in laboratory rats [104] and mice [105] with the basal-apical zoning of the vomeronasal sensory epithelium. That is, neurons projecting to the anterior part of the AOB locate their somas in the apical part of the vomeronasal sensory neuroepithelium, while those projecting to the posterior zone are positioned basally. However, this basal-apical zonation has not been described in other rodents in which G protein expression in the VNO has been studied, such as in the case of the fossorial water vole [41], the beaver [39], and the capybara [36], or even in mammals belonging to the segregated model, that is, those that exhibit anteroposterior zoning in the AOB, such as the rabbit [36] or the wallaby [28]. It is remarkable that the zonation found with lectins in the AOB of the fossorial water vole corresponds to the labelling pattern observed with UEA, LEA, and STA lectins. That is, the V1R axons reaching the anterior part of the AOB share a selective and analogous expression of glycoconjugates, which is different from that present in the V2R axons reaching the posterior part of the AOB.

Calcium-binding proteins exhibited in the AOB characteristic patterns indicating high activity in significant neuronal subpopulations within this region. Calbindin and calretinin revealed the laminated structure of the AOB. Specifically, both CB and CR produced dense and intense labelling in the nerve and glomerular layers of the AOB. In the mitral and granular layers, there was also intense labelling of the neuropil and cell bodies, although this was more pronounced in the case of calretinin. In the MOB, both antibodies produced a similar pattern, with dense labelling in the nerve and glomerular layers, but without staining the glomerular neuropil, concentrating exclusively in periglomerular cells. This pattern was similar to that described with anti-CR in the AOB and MOB of the rat [106]. Differences in CB labelling were more pronounced across species within the superficial layers of the AOB compared to CR labelling. In particular, studies on rats [107] and opossums [108,109] demonstrated that the neuropil displayed notably less staining than observed in fossorial water voles. Nonetheless, specific individual glomeruli in both rats and opossums displayed immunostaining and were associated with occasionally positively stained PG cells.

A remarkable finding was the identification with both anti-CB and anti-CR of a significant cluster of neurons located in the caudal sub-bulbar area. The role of these neuronal groups remains unknown, but their proximity to the AOB suggests they may be involved in the detection of chemical signals. To our knowledge, similar nuclei have only been described in the rat [110], hamster [111], rabbit [29,112], and hedgehog [113], but in all cases without the use of neuromarkers such as calbindin (CB) or calretinin (CR). Additionally, anti-calretinin strongly labelled the rostral migratory stream. Another calcium-binding protein studied was secretagogin, although in this case the labelling pattern was more diffuse and less distinct, likely due to generalized labelling of the neuropil. Nevertheless, the presence of immunopositive periglomerular and mitral cells of the AOB was noticeable.

The antibodies against MAP-2 and GAP-43 produced intense and revealing immunopositivity in the AOB of the fossorial water vole. Anti-MAP-2 is an excellent marker for the dendritic trees of mitral and granular cells [114], but it does not stain their somas or axons [115]. This is reflected in strong anti-MAP-2 immunolabelling in both the mitral-plexiform and granular layers, accompanied by immunonegativity in the somas of the mitral and granular cells of the AOB and LOT. Although MAP-2 immunolabelling should reveal the contribution of mitral cells to the glomeruli, as observed in the mouse AOB [116], in the case of the fossorial water vole, this contribution is fainter.

GAP-43 serves as an effective marker for distinguishing between mature axons and regenerating nerve fibers, as its expression quickly diminishes in newly developed fibers once they have arrived at their destinations [67,117]. As it has been previously described in rabbits [29], the immunohistochemical labelling of GAP-43 in the fossorial water vole is a useful probe for discriminating the external and internal plexiform layers of the MOB, as well as the contribution of both plexiform layers to the mitral-plexiform layer of the AOB. According to GAP-43 immunostaining, we can conclude that the existence of two plexiform layers in the AOB is far from a theoretical concept.

The antibody against GAP-43 did not reveal the zonal organization of the AOB, as was exceptionally demonstrated in rabbits [29]. However, a remarkable observation—and, to our knowledge, specific to the fossorial water vole—is the strong immunostaining of the somas of the mitral cells in the AOB, as well as the mitral and tufted cells of the MOB with anti-GAP-43. In adult neurons, high levels of GAP-43 expression are often linked to ongoing synaptic remodeling or heightened plasticity [118]. In the case of the fossorial water vole, a subterranean animal that relies heavily on its sense of smell due to limited vision, the olfactory system may require enhanced adaptability to effectively process environmental cues [119]. The increased GAP-43 expression in mitral and tufted cells could reflect a higher degree of synaptic plasticity, necessary for refining olfactory and pheromonal processing under the unique conditions of an underground habitat. This adaptation might be crucial for activities such as foraging, navigation, and social interactions, where olfactory and semiochemical cues play a significant role. Further research would be necessary to confirm this hypothesis and to understand the specific roles of GAP-43 in the olfactory system of fossorial water voles.

Olfactory Marker Protein (OMP) is recognized as a specific marker for the superficial layers throughout the entire olfactory bulb. In the fossorial water vole, OMP immunostaining is confined to the nervous strata and glomerular layers, exhibiting a slightly weaker intensity in the AOB compared to the main olfactory bulb, the MOB. The expression of OMP is typical of mature neurons that carry olfactory and vomeronasal receptors [120,121]. Although OMP expression has been widely studied across various mammalian VNOs [46,122], investigations into its immunoreactivity within the AOB have been limited to certain species. These include rodents like mice, rats, and hamsters [94,123]. While the exact function of OMP is yet to be fully elucidated, it is hypothesized to play a role in the maturation of olfactory and vomeronasal neurons [124]. Moreover, OMP is implicated in the formation and refinement of the glomerular map, contributing to the precise organization of neural connections within the olfactory bulb [125]. Correspondingly, OMP labelling has been observed to increase with age in both the VNO and the AOB [67,126].

Protein gene product 9.5 (PGP 9.5) is a soluble protein initially isolated from human brain tissue. It corresponds to the enzyme ubiquitin carboxyl-terminal hydrolase, which recycles ubiquitin from ubiquitin-linked protein complexes or polyubiquitin chains involved in proteolytic pathways [127]. Previous research has identified the expression of PGP 9.5 in both developing and mature olfactory and vomeronasal receptor cells of rats [69,128]. Moreover, studies have shown that PGP 9.5 predominantly localizes in the mitral and tufted cells within the main and accessory olfactory bulbs of rats and hamsters [43,69]. In this study, we confirmed the widespread immunoreactivity of PGP 9.5 in the accessory olfactory bulb of the fossorial water vole, with significant labelling in the mitral cells of the mitral-plexiform layer as well as in periglomerular and short-axon cells within the glomerular layer. This distribution suggests that PGP 9.5 is essential for sustaining high levels of neuronal activity, critical for olfactory processing.

The immunohistochemical labelling with anti-SMI32 emphasizes the differences between the AOB and MOB in terms of neuronal activity and synaptic organization. The absence of anti-SMI32 labelling in the AOB, contrasted with strong labelling in the MOB, provides further evidence of the differential roles that these two structures play in olfactory signal processing. SMI32 is a marker for non-phosphorylated neurofilaments associated with mature neurons [129], which may indicate that the MOB handles more direct and rapid signal transmission, whereas the AOB might be involved in processing more complex, integrative signals related to social behaviors. Finally, the identification of active neurogenesis within the rostral migratory stream (RMS), immunolabelled with anti-DCX, is consistent with the known capacity for continuous neuronal turnover in the olfactory bulb [130]. This reflects a dynamic process of neural regeneration in the olfactory system of the fossorial water vole, likely a crucial adaptation for maintaining olfactory sensitivity in the context of a changing subterranean environment.

## 5. Conclusions

In conclusion, our study underscores the structural and functional complexities of the AOB in rodents. The fossorial water vole, with its distinct reliance on chemical senses, provides an excellent model for understanding the neuroanatomical adaptations of the olfactory system in subterranean species. Future research should focus on exploring the functional consequences of the observed structural differences, particularly in the context of kairomonal and pheromonal detection, which could offer insights into the development of pest control strategies targeting chemical communication pathways [59,60,131].

## Figures and Tables

**Table 1:** Detailed information on the antibodies used in this study: species of elaboration, dilution, catalogue number, manufacturer, target immunogens, and secondary antibody employed.

Antibody	1st Ab Species	Dilution	Supplier/ Catalog Number	Target Immunogen	2nd Ab Species/Catalog Number
Anti-Gαo	Rabbit	1:200	MBL-551	Bovine GTP binding protein Gαo subunit	ImmPRESS VR HRP Anti-rabbit IgG Reagent MP-6401-15
Anti-Gαi2	Rabbit	1:100	Santa Cruz Biotech. SC-7276	Peptide mapping within a highly divergent domain of Gαi2 of rat origin	ImmPRESS VR HRP Anti-rabbit IgG Reagent MP-6401-15
Anti-CB	Rabbit	1:6000	Swant-CB38	Rat recombinant calbindin D-28K	ImmPRESS VR HRP Anti-rabbit IgG Reagent MP-6401-15
Anti-CR	Rabbit	1:6000	Swant-7697	Recombinant human calretinin containing a 6-His tag at the N-terminus	ImmPRESS VR HRP Anti-rabbit IgG Reagent MP-6401-15
Anti-MAP-2	Mouse	1:200	Sigma M4403	Rat brain microtubule-associated proteins	ImmPRESS VR HRP Anti-mouse IgG Reagent MP-6402-15
Anti-GAP-43	Mouse	1:800	Sigma G9264	HPLC-purified GAP43 from neonatal rat forebrain	ImmPRESS VR HRP Anti-mouse IgG Reagent MP-6402-15
Anti-OMP	Mouse	1:200	Santa Cruz Biotech. SC-365818	Amino acids 1-163 of the total human-origin OMP	ImmPRESS VR HRP Anti-mouse IgG Reagent MP-6402-15
Anti-DCX	Rabbit	1:300	Proteintech 13925-1-AP	DCX fusion protein Ag4945	ImmPRESS VR HRP Anti-rabbit IgG Reagent MP-6401-15
Anti-SMI-32	Rabbit	1:20	Enzo ABS-219-0100	Nonphosphorylated neurofilaments from rat brain	ImmPRESS VR HRP Anti-rabbit IgG Reagent MP-6401-15
Anti-PGP	Rabbit	1:200	Proteintech 14730-1-AP	UCHL1/PGP 9.5 fusion protein Ag6490	ImmPRESS VR HRP Anti-rabbit IgG Reagent MP-6401-15
Anti-SG	Rabbit	1:400	Gift from L Wagner (University of Vienna, Austria)	Recombinant human secretagogin	ImmPRESS VR HRP Anti-rabbit IgG Reagent MP-6401-15

Abbreviations: CB, calbindin; CR, calretinin; DCX, doublecortin; GAP-43, growth-associated protein-43; HRP, horseradish peroxidase; MAP-2, microtubule-associated protein-2; OMP, olfactory marker protein; PGP 9.5, protein gene product 9.5; SG, secretagogin; SMI-32, neurofilament protein SMI-32.

**Table 2:** Lectins used, concentrations, and binding specificities.

Lectin	Abbreviation	Dilution (mg/mL)	Supplier Catalog Number	Preferred Sugar Specificity	Specificity Groups
*Ulex europaeus* (Gorse) agglutinin	UEA	2.0	Vector B-1065-2	α-Fuc	Fucose
*Lycopersicon esculentum* (Tomato) lectin	LEA	1.0	Vector B-1175-1	β-1,4 GlcNAc oligomers	GlcNAc
*Vicia villosa* (Hairy Vetch) agglutinin	VVA	2.0	Vector B1235-2	GalNAc	Gal/GalNAc
*Solanum tuberosum* (Potato) lectin	STL	2.0	Vector B-1165-2	GlcNAc Oligomers, LacNAc	GlcNAc
*Dolichos biflorum* (Horse gram) lectin	DBA	2.0	Vector B-1035-5	α GalNAc	GalNAc

Abbreviations: Fuc, fucose; Gal, galactose; GalNAc, N-acetyl-galactosamine; GlcNAc, N-acetyl-glucosamine; LacNac, N-acetyl-lactosamine.

## Data Availability

The original contributions presented in the study are included in the article, further inquiries can be directed to the corresponding author/s.
